# Identification of Dezidougou Virus in a DAK AR 41524 Zika Virus Stock

**DOI:** 10.1128/genomeA.00605-17

**Published:** 2017-07-27

**Authors:** Susmita Shrivastava, Vinita Puri, Nadia Fedorova, Paolo Amedeo, Timothy B. Stockwell, Reed S. Shabman, Sujatha Rashid, Brett E. Pickett

**Affiliations:** a J. Craig Venter Institute, Rockville, Maryland, USA; b BEI Resources, Manassas, Virginia, USA; c J. Craig Venter Institute, La Jolla, California, USA

## Abstract

We report here the complete genome of a Dezidougou virus (DEZV) isolated from a passaged culture of the Zika virus strain DAK AR 41524. The consensus DEZV sequence we recovered shows 99% nucleotide similarity using BLASTN to a previously reported DEZV (accession no. JQ675604.1). The current sequence has additional repeat regions as well as a deleted repeat region, which we confirmed by Sanger sequencing, that were not present in the originally published sequence, JQ675604.1.

## GENOME ANNOUNCEMENT

Dezidougou virus (DEZV) is a single-stranded, monopartite, positive-sense RNA virus of approximately 9.3 kb in length, and it is a member of the recently proposed *Negevirus* taxon. Here, we report the most complete sequence for a Dezidougou virus available to date, which is missing only the start of the coding region. This virus was obtained from a passage of Zika virus (ZIKV) strain DAK AR 41524, which was isolated from a mosquito (*Aedes africanus*) in Kédougou, Senegal, on 17 November 1984. This isolate was passaged twice in mosquito cell lines, first in AP61 (*Aedes pseudocutellaris*) and then in C6/36 (*Aedes albopictus*) cells at the World Reference Center for Emerging Viruses and Arboviruses (WRCEVA). The isolate was then passaged twice in C6/36 cells, where it showed good cytopathic effect but failed subsequent molecular authentication for Zika virus. In parallel, the seed Zika virus from WRCEVA that was passaged in the Vero cell line (African green monkey) grew to high titers. Viral RNA was extracted from the supernatant of mosquito cell cultures and subjected to sequence-independent single-primer amplification, as described previously (1). Next-generation sequencing libraries were then constructed, and the samples were sequenced on the Illumina MiSeq platform (2 × 300 bp). This approach enabled unbiased identification of the Dezidougou virus strain from a mosquito cell line.

Sequencing reads were demultiplexed by barcode, trimmed, and then *de novo* assembled. BLASTN searches of these contigs were then performed against the GenBank nonredundant nucleotide database (2). Following genome assembly, raw reads were mapped to both the ZIKV and DEZV reference genomes. In total, 53,868 reads were obtained for Dezidougou virus, while only 1,376 reads were obtained for the DAK AR 41524 Zika strain. Separate reference-based assemblies were generated, using both ZIKV and Dezidougou virus (accession no. JQ675604.1). No differences were found between the DEZV consensus sequence obtained from *de novo* assembly compared against the assembly obtained by using the new strain as a reference.

The presence of DEZV in the mosquito-derived culture confirms previous results that negeviruses replicate to high titer only in mosquito cells and do not appear to replicate in mammalian cells or mice (3). It is therefore likely that this DEZV strain has been present in the ZIKV sample since the initial isolation from the mosquito source. To our knowledge, the coisolation of genetic material from DEZV and ZIKV from the same virus stock has not been previously described.

After comparing our consensus to the publically available Dezidougou virus sequence (JQ675604.1), we observed one 396-base insertion beginning at position 6714 in JQ675604.1. The 201-base duplication found in JQ675604.1 at position 8780 was not detected in the J. Craig Venter Institute (JCVI) consensus sequence. These duplications were then verified with Sanger sequencing technology. Sequences of additional DEZV strains will confirm the true consensus and the genomic structure of Dezidougou virus.

### Accession number(s).

The consensus sequence for DEZV was manually annotated and deposited in GenBank with the accession number KY968698 and BioProject ID PRJNA350806.
